# An Assessment of Deep Learning’s Impact on General Dentists’ Ability to Detect Alveolar Bone Loss in 2D Intraoral Radiographs

**DOI:** 10.3390/diagnostics15040467

**Published:** 2025-02-14

**Authors:** Amjad AlGhaihab, Antonio J. Moretti, Jonathan Reside, Lyudmila Tuzova, Donald A. Tyndall

**Affiliations:** 1 Department of Maxillofacial Surgery and Diagnostic Sciences, College of Dentistry, King Saud bin Abdulaziz University for Health Sciences, Riyadh 11481, Saudi Arabia; 2 King Abdullah International Medical Research Center, Ministry of National Guard Health Affairs, Riyadh 11481, Saudi Arabia; 3 Department of Diagnostic Sciences, Oral and Maxillofacial Radiology, Adams School of Dentistry, University of North Carolina at Chapel Hill, Chapel Hill, NC 27599, USA; 4 Department of Periodontology, Endodontics and Dental Hygiene, Adams School of Dentistry, University of North Carolina at Chapel Hill, Chapel Hill, NC 27599, USA; 5 Denti.AI Technology Inc., Toronto, ON M5R 3K5, Canada

**Keywords:** alveolar bone loss, artificial intelligence, deep learning, dental digital radiography

## Abstract

**Background/Objective:** Deep learning (DL) technology has shown potential in enhancing diagnostic accuracy in dentomaxillofacial radiology, particularly for detecting carious lesions, apical lesions, and periodontal bone loss. However, its effect on general dentists’ ability to detect radiographic bone loss (RBL) in clinical practice remains unclear. This study investigates the impact of the Denti.AI DL technology on general dentists’ ability to identify bone loss in intraoral radiographs, addressing this gap in the literature. **Methods:** Ten dentists from the university’s dental clinics independently assessed 26 intraoral radiographs (periapical and bitewing) for bone loss using a Likert scale probability index with and without DL assistance. The participants viewed images on identical monitors with controlled lighting. This study generated 3940 data points for analysis. The statistical analyses included receiver operating characteristic (ROC) curves, area under the curve (AUC), and ANOVA tests. **Results:** Most dentists showed minor improvement in detecting bone loss on periapical radiographs when using DL. For bitewing radiographs, only a few dentists showed minor improvement. Overall, the difference in diagnostic accuracy between evaluations with and without DL was minimal (0.008). The differences in AUC for periapical and bitewing radiographs were 0.031 and −0.009, respectively, and were not statistically significant. **Conclusions:** This study found no statistically significant improvement in experienced dentists’ diagnostic accuracy for detecting bone loss in intraoral radiographs when using Denti.AI deep learning technology.

## 1. Introduction

Artificial intelligence (AI) is a field within computer science aimed at enabling machines to perform tasks requiring human intelligence, such as learning, reasoning, and decision-making [1]. Within AI, machine learning (ML) is a pivotal subset in which algorithms iteratively learn from data without explicit programming, improving over time as more information is analyzed [2]. Deep learning (DL), a branch of ML, utilizes deep neural networks inspired by neurons in the human brain, enabling computers to recognize complex patterns and execute intricate tasks. These networks consist of layered, interconnected neurons that process data, adjust connections through learning algorithms, and adapt over time. Different types of neural networks have been developed, each with a distinct underlying architecture designed to process specific types of data, leading to a variety of applications. Convolutional neural networks (CNNs) are particularly suited for tasks involving image processing, recognition, and classification due to their spatial hierarchies of features [1,3].

The integration of DL in dentistry is revolutionizing the field by providing a reliable “second opinion,” thus enhancing diagnostic accuracy, treatment planning, and patient care [4]. Numerous studies have demonstrated the efficacy of DL in various diagnostic tasks, including tooth detection and numbering [5]; orthodontic landmark localization [6]; temporomandibular joint assessment [7]; and the identification of dental caries [8], periapical lesions [9], and periodontal bone loss [10,11,12,13,14]. Additionally, DL is aiding in the development of personalized treatment plans and recommending optimal treatment strategies [15,16]. By leveraging the capabilities of deep learning, the field of dentistry is experiencing significant advancements in both clinical and operational aspects, ultimately leading to improved patient outcomes and streamlined dental care processes [16].

Denti.AI (Denti.AI Technology Inc., Toronto, ON, Canada) (Version 1.3 http://denti.AI, accessed on 6 November 2023), an FDA-cleared and commercially available software, utilizes DL to detect carious lesions, apical lesions, and radiographic bone levels [17]. Its regulatory approval underscores its potential clinical relevance, emphasizing the need to evaluate its impact on enhancing diagnostic accuracy among general dentists during routine clinical practice. Testing the clinical impact of such software is crucial. Notably, only one study to date has assessed this software’s effectiveness among general dentists and residents, demonstrating improved diagnostic performance in detecting apical radiolucencies on periapical radiographs when using the software [18].

To the best of our knowledge, no studies in the English literature have specifically evaluated the impact of DL on general dentists’ diagnostic performance in detecting radiographic bone loss (RBL). The existing research has largely concentrated on assessing the standalone accuracy of DL technology rather than its practical utility in clinical settings [10,11,12,13,14]. Therefore, this study aims to fill that gap by exploring how DL technology affects general dentists’ ability to identify bone loss in intraoral radiographs, providing insights into its potential value in everyday dental practice.

## 2. Materials and Methods

### 2.1. Observer Recruitment and Intraoral Radiograph Selection

This study was approved by the university’s Institutional Review Board (IRB 21-3251). An email outlining this study was distributed to all general dentists (*n* = 17) at the university in January 2024. A recruitment period of three months was established, allowing participation from January to March. Ten general dentists, each with over five years of clinical experience, volunteered to take part in this study. Verbal informed consent was obtained from all the participants.

**Figure 1:**
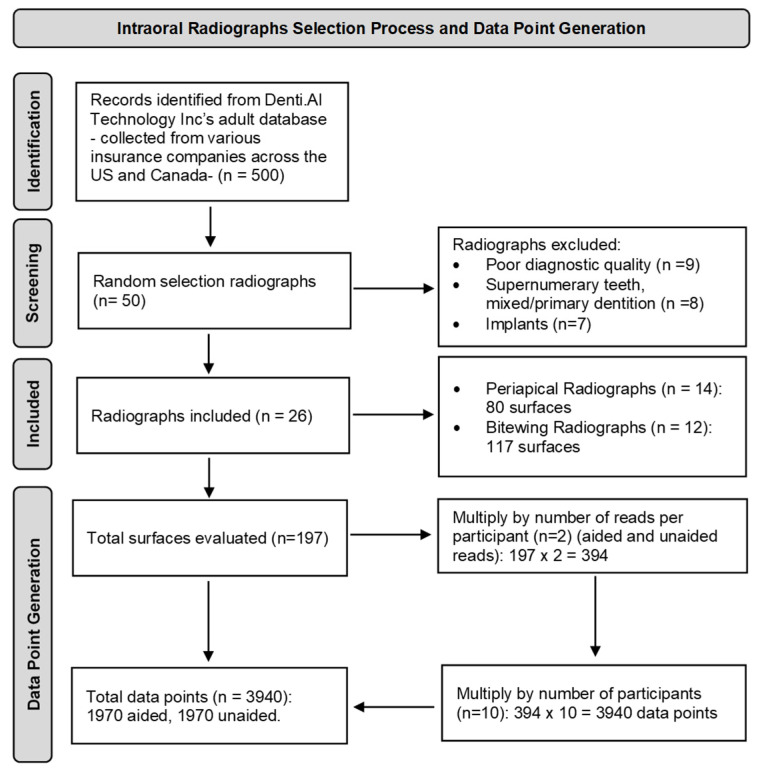
Flowchart illustrating the selection process for the intraoral radiographs and tooth surfaces. A total of 26 intraoral radiographs (12 bitewing radiographs and 14 periapical radiographs) were selected, with each radiograph evaluated for radiographic bone loss (RBL) on multiple tooth surfaces. Positive and control surfaces were identified, generating a total of 3940 data points for analysis, capturing assessments with and without the use of deep learning (DL) assistance.

The primary investigator (AA) accessed a large pool of de-identified intraoral radiographs provided by Denti.AI (Denti.AI Technology Inc., Toronto, ON, Canada), sourced from various insurance companies over the past five years across the United States (U.S.) and Canada. The radiographs were collected randomly by the company, and the primary investigator randomly selected 50 radiographs. Of these, 26 intraoral radiographs (14 periapical radiographs and 12 bitewing radiographs) were chosen for this study based on inclusion criteria ensuring they were diagnostically acceptable and represented a diverse range of cases to evaluate the diagnostic performance of the general dentists. Radiographs of poor diagnostic quality and those showing primary/mixed dentition, supernumerary teeth, or implants were excluded (Figure 1). The reporting of this study followed the Standards for Reporting Diagnostic Accuracy Studies (STARD) 2015 guidelines to ensure completeness and transparency in the presentation of diagnostic accuracy outcomes [19].

### 2.2. Calibration and Clinical Performance Testing

The participants attended a calibration session using practice cases that were not included in the actual study. During this session, they completed a comprehensive practice assessment by evaluating 10 intraoral radiographs (five periapical radiographs and five bitewing radiographs), covering a total of 34 teeth and 55 tooth surfaces. This hands-on practice was designed not only to familiarize participants with the software interface but also to ensure consistency in applying the threshold criteria used to define RBL, as described in the subsequent section. The calibration aimed to minimize variability in the assessments, thereby improving the reliability of the study results.

Following the calibration session, each participant independently evaluated 26 intraoral radiographs (14 periapical radiographs and 12 bitewing radiographs) for alveolar bone loss, covering a total of 120 teeth and 197 tooth surfaces (80 surfaces in periapical radiographs and 117 surfaces in bitewing radiographs). All the participants viewed the images on identical monitors under controlled lighting conditions. Selected tooth surfaces were first scored without, and subsequently with, the use of DL technology in the same session. A Likert scale probability index (PRI) [20] ranging from 1 to 5, was employed to assess both the presence of alveolar bone loss and the clinician’s confidence in their assessment of each radiograph. The scores were defined as follows: 1 = definitely no radiographic bone loss, 2 = probably no radiographic bone loss, 3 = unsure, 4 = probably radiographic bone loss, and 5 = definitely radiographic bone loss. This process generated a total of 3940 data points for analysis, with 1970 data points collected without DL technology and 1970 with DL technology (Figure 1).

### 2.3. The Deep Learning Software, Definition of RBL Used, and Reference Standard

This study employed deep learning software from Denti.AI (Denti.AI Technology Inc., Toronto, ON, Canada) (Version 1.3, http://denti.ai, accessed on 6 November 2023) [17], featuring multiple models utilizing CNN frameworks. In this software, teeth are detected via a faster region-based CNN (RCNN), which draws bounding boxes, followed by classification and numbering using the residual network (ResNet) CNN. For bone-level keypoints, the model processes the radiograph, identifies tooth bounding boxes, extracts features, and assigns keypoints using ResNet CNN and feature pyramid network (FPN) architectures with two heads: one for keypoint coordinates and another for confidence. The bone-level detection module predicts anatomical landmarks on the mesial and distal sides of each tooth—referred to as “surfaces” in this study—identifying the cementum–enamel junction (CEJ), bone level, and root apex, along with confidence probabilities for each landmark. These landmarks are used to measure bone levels, calculating distances from the CEJ to the bone and from the CEJ to the root in millimeters and automatically determining the ratios of the CEJ–bone to CEJ–root distances.

**Figure 2:**
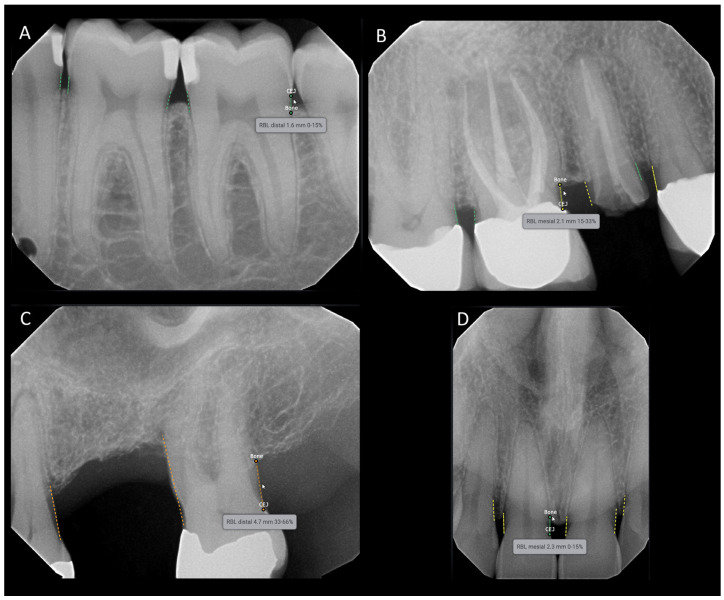
Example of the Denti.AI software interface predicting radiographic alveolar bone loss for periapical radiographs. The software detects and color codes the measurements: green for 0–15% (**A**,**D**), yellow for 15–33% (**B**), and orange for 33–66% (**C**). The measurements are displayed when the mouse is dragged over the area of interest.

**Figure 3:**
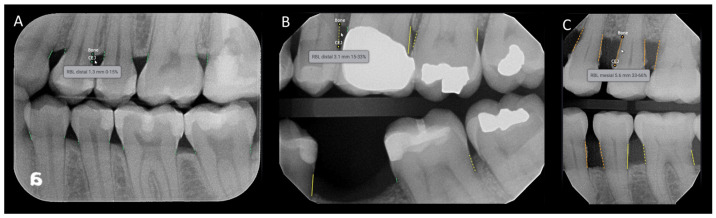
Example of the Denti.AI software interface predicting radiographic alveolar bone loss for bitewing radiographs. The software detects and color codes the measurements: green for 0–15% (**A**), yellow for 15–33% (**B**), and orange for 33–66% (**C**). The measurements are displayed when the mouse is dragged over the area of interest.

The annotated landmarks were used to measure bone levels and identify the presence of RBL. The criteria for RBL were based on the periodontitis staging and grading guidelines from the 2017 World Workshop by the American Academy of Periodontology (AAP) and the European Federation of Periodontology (EFP) [21]. In bitewing radiographs, RBL is present if the distance between the CEJ and the most apical part of the alveolar bone is 2 mm or more. In periapical radiographs, RBL is determined by the ratio of alveolar bone loss to the total root length of the tooth, expressed as a percentage. RBL is considered present if this percentage is 15% or higher. Table 1 provides an overview of the sampled intraoral radiographs categorized according to the AAP/EFP guidelines. The Denti.AI software (Denti.AI Technology Inc., Toronto, ON, Canada) (Version 1.3, http://denti.ai, accessed on 6 November 2023)detects and color codes the measurements: green for 0–15%, yellow for 15–33%, orange for 33–66%, and red for >66% (Figure 2 and Figure 3). For the statistical analysis, each case was ultimately classified in a binary system in which RBL was denoted as present or absent.

The reference standard for RBL was established by a consensus panel consisting of a board-certified oral and maxillofacial radiologist and two periodontists. The panel annotated all the radiographs used in this study, identifying the same key landmarks and guidelines for measuring and detecting RBL, respectively. The interobserver agreement between the two periodontists was assessed using the Kappa statistic, yielding values of 0.69 for periapical radiographs and 0.83 for bitewing radiographs, indicating substantial and almost perfect agreement, respectively [22]. The oral and maxillofacial radiologist then finalized the determinations regarding the presence or absence of RBL, enhancing the robustness of the reference standard. Prior to observer testing, the deep learning software was validated against the reference standard, with results from this standalone evaluation awaiting publication. For the observer study, the Denti.AI software’s (Denti.AI Technology Inc., Toronto, ON, Canada) (Version 1.3, http://denti.ai, accessed on 6 November 2023)’s annotations were verified to align with those of the consensus panel, ensuring a reliable benchmark for assessing the diagnostic performance of the general dentists.

### 2.4. Statistical Analysis

The intended sample size for the observers included 17 general dentists, of whom 10 agreed to participate. This sample size was determined based on the availability of qualified dentists at the university and the anticipated feasibility of participation. Estimating the sample size in AI studies is challenging and typically requires a pilot study to measure observer improvement [20]; this study provides initial valuable data on AI’s impact magnitude on observers’ performance. The RJafroc R-library (version 2.0.1, https://dpc10ster.github.io/RJafroc/index.html, (accessed on 6 November 2023)) was used to assess the clinicians’ performance [23]. Receiver operating characteristic (ROC) curves and area under the curve (AUC) were used to evaluate the performance of the binary classification system. ANOVA-based methods for multi-reader multi-case analysis assessed the significance of clinicians’ performance shifts with and without the use of AI support. The Dorfman–Berbaum–Metz (DBM) method was used with a random-reader random-case (RRRC) analysis option, treating both readers and cases as random factors. Statistically significant results, indicated by a P-value of less than 0.05, suggest that the findings can be generalized to the population of readers and cases [24].

## 3. Results

**Figure 4:**
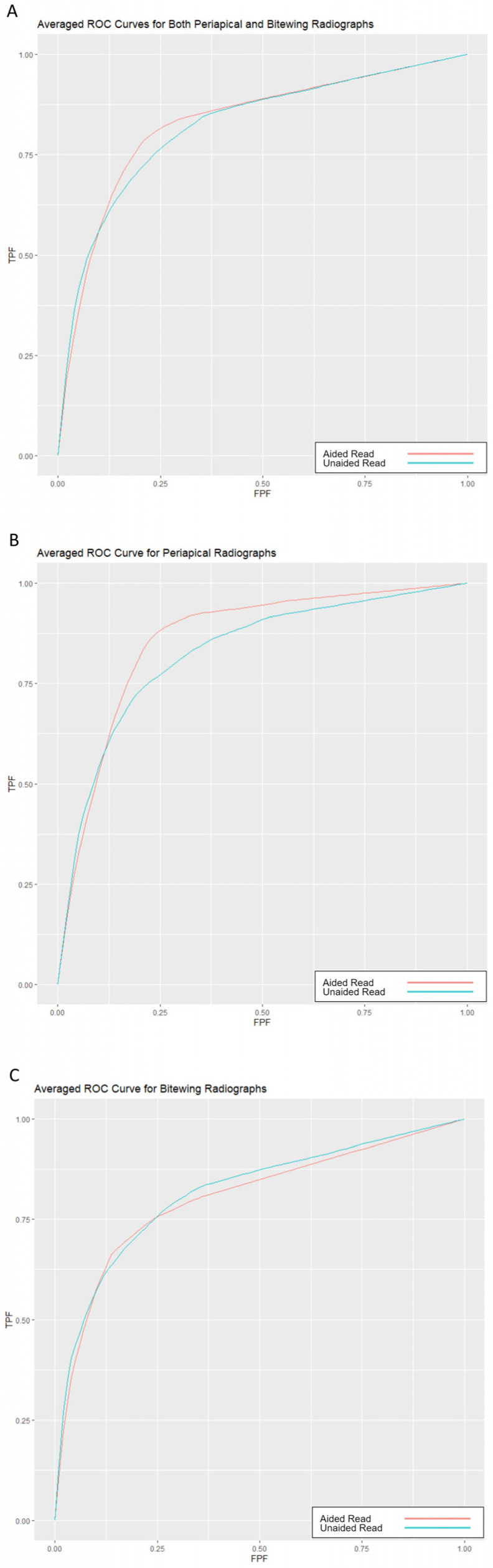
Averaged receiver operating characteristic (ROC) curves and corresponding area under the curve (AUC) for the ten readers across two reading modes: unaided (blue) and aided (red). The curves represent the relationship between the true positive fraction (TPF or sensitivity) and the false positive fraction (FPF or 1-specificity). (**A**): Averaged ROC curve for both periapical and bitewing radiographs combined. The AUC is 0.823 for the unaided mode and 0.830 for the aided mode. (**B**): Averaged ROC curve for periapical radiographs. The AUC is 0.831 for the unaided mode and 0.862 for the aided mode. (**C**): Averaged ROC curve for bitewing radiographs. The AUC is 0.821 for the unaided mode and 0.812 for the aided mode.

Eight, out of the ten general dentists, showed a minor improvement in alveolar bone loss detection on periapical radiographs, when using DL. However, for bitewing radiographs, only three out of the ten general dentists showed a minor improvement. Overall, the difference in diagnostic accuracy (AUC) between evaluations with and without DL was minimal (0.008). When analyzing periapical and bitewing radiographs separately, the differences in AUC were 0.031 and −0.009 for periapical and bitewing intraoral radiographs, respectively. For all the cases, the differences were not statistically significant (Table 2 and Figure 4).

## 4. Discussion

Although most research has focused on evaluating the standalone accuracy of DL technology in detecting RBL, no studies, to date, have assessed its practical utility when integrated into the workflow of general dentists. In modern clinical practice, understanding how such technology impacts daily diagnostic performance is essential, as it provides insights beyond controlled settings and highlights its true value for patient care. The findings of the current study did not reveal a significant enhancement in the diagnostic accuracy of general dentists for detecting RBL in periapical and bitewing radiographs when aided by DL. Although statistical significance was not observed for either type of radiograph, certain trends were observed. In the periapical radiographs, most observers showed a minor improvement in diagnostic tasks when utilizing DL, whereas, in the bitewing radiographs, only a few observers demonstrated a minor improvement. This may be because bitewing geometry is more favorable for bone-level assessment than periapical radiographs, which are more prone to geometric distortions. The slight improvement for periapical radiographs likely reflects these geometric constraints, absent in most bitewings. In other words, bitewing radiographs are generally more accurate and easier to assess than periapical radiographs, resulting in no improvement using AI, whereas AI may be more beneficial for assessing bone levels in periapical radiographs. Several factors may account for the lack of statistical significance observed in this study.

Firstly, the absence of a time limit during the experiment could have influenced the outcomes. This setting does not reflect everyday clinical scenarios when time constraints are common. It is hypothesized that AI could potentially improve efficiency by reducing time spent in clinical practice, Hegde et al. (2023) conducted a systematic review identifying time pressure as a contributing factor to errors in interpreting dental radiographs, highlighting its impact on diagnostic accuracy [25]. Similarly, Plessas et al. (2019) investigated the effect of time pressure on dentists’ diagnostic performance in interpreting bitewing radiographs, finding a correlation with decreased sensitivity and accuracy, thus increasing interpretive errors [26].

Furthermore, the clinical experience of the observers may have influenced the study outcomes. The majority of participants were general dentists with over five years of experience. If this study had included senior dental students or recent graduates, there might have been statistically significant differences in diagnostic performance with and without AI. Hegde et al. (2023) concluded that clinicians with less experience tend to exhibit higher rates of interpretive errors [25]. So, it is possible that the advanced clinical experience of the participants mitigated the observable impact of AI assistance, as experienced clinicians may already achieve a high baseline accuracy, reducing the relative contribution of AI support.

Additionally, performance bias may have played a role in the lack of statistically significant findings. Research often overestimates observer performance due to participants performing better when they are aware of being evaluated, a phenomenon known in the literature as the Hawthorne effect. This psychological concept highlights how evaluation awareness can prompt behavioral changes in research participants [27]. McCambridge et al. (2014) conducted a systematic review focused on the Hawthorne effect. The authors argued that traditional interpretations of the Hawthorne effect may oversimplify complex dynamics of research participation, suggesting a need for new conceptual frameworks to better understand how the awareness of being studied influences participant behavior and study outcomes. Their review underscored the importance of nuanced approaches in studying the effects of research participation to enhance the validity and interpretation of findings across various fields of research [28].

According to Yu et al. (2024), while AI assistance holds promise in enhancing radiologists’ diagnostic accuracy, there are instances in which its integration can paradoxically worsen their performance. Factors contributing to this include overreliance on AI recommendations leading to cognitive biases, misinterpretation of AI-generated data, and/or inadequate training in AI utilization. This study highlights that when dental clinicians excessively trust AI outputs without critical evaluation or lack the skills to integrate AI insights effectively, diagnostic errors can occur, potentially compromising patient care. Thus, understanding the nuances of AI’s impact and ensuring appropriate training and guidelines are crucial to harnessing its benefits while mitigating potential drawbacks in clinical settings [29].

While the potential drawbacks of AI integration in clinical settings warrant careful consideration, it is equally important to acknowledge AI’s ability to enhance workflow efficiency, even in the absence of significant improvements in diagnostic accuracy. By pre-analyzing radiographs and flagging areas of concern, AI can reduce the time required for manual assessments, allowing clinicians to focus on confirming or refining AI-suggested findings. This can streamline the diagnostic process and free up valuable time for other clinical responsibilities. Additionally, AI offers a standardized approach to pre-screening, minimizing variability among clinicians and ensuring consistency in routine cases. For less experienced practitioners, AI can serve as a valuable training aid, guiding them through complex cases while enhancing their confidence and decision-making skills. These workflow benefits highlight AI’s ability to optimize resource use and improve overall efficiency, even when the diagnostic accuracy remains unchanged [30].

The current study has several limitations. Firstly, the sample size of 10 participants may have been insufficient to achieve the statistical power needed to detect significant differences, limiting the generalizability of the findings. Although no statistically significant improvements were observed, this does not rule out the presence of an effect. The small effect size observed suggests that a larger sample of general dentists might be required to identify significant differences. Additionally, the absence of a time constraint may not fully reflect daily routine clinical conditions, potentially influencing the clinicians’ diagnostic behavior and outcomes. Lastly, the observers in this study had more than five years of clinical experience, resulting in limited variation in clinical expertise, which may have minimized the potential impact of AI assistance on the diagnostic performance. To strengthen the evidence, future studies should aim to include a larger sample size, incorporate participants with varying levels of clinical experience, and test under more realistic clinical conditions, including time constraints, to more accurately assess the practical utility of DL technology.

## 5. Conclusions

This study did not find a statistically significant improvement in the clinical diagnostic accuracy of experienced dentists for detecting RBL in intraoral radiographs when utilizing Denti.AI DL technology.

## Figures and Tables

**Table 1:** An overview of the radiograph samples with and without radiographic bone loss (RBL) based on AAP/EFP guidelines ^1^.

Threshold	<15% (Control Surfaces)	Positive Surfaces	
		15–33%	33–66%
Periapical radiograph (*N* = 14 with 80 surfaces)	40	32	8
**Threshold**	**<2 mm (Control Surfaces)**	**Positive Surfaces**	
		**2–4 mm**	**≥5 mm**
Bitewing radiograph (*N* = 12 with 117 surfaces)	58	49	10

^1^ Based on AAP/EFP guidelines, in bitewing radiographs, RBL is considered present if the CEJ–bone distance is ≥2 mm and, in periapical radiographs, if alveolar bone loss is ≥15% of root length [21]. For statistical purposes, each case was categorized in a binary system: cases with RBL were marked as “positive surfaces” and those without as “control surfaces”.

**Table 2:** Receiver operating characteristic curves and area under the curve for the ten readers before and after deep learning utilization.

All Data											
	Estimate	*p*-Value	CILB	CIUB							
ROC AUC Before DL–After DL	0.008	0.725	−0.037	0.053							
	R1	R2	R3	R4	R5	R6	R7	R8	R9	R10	Averaged ROC AUC
Before DL	0.807	0.854	0.828	0.848	0.814	0.889	0.791	0.867	0.719	0.809	0.823
After DL	0.876	0.875	0.816	0.843	0.822	0.803	0.742	0.855	0.819	0.852	0.830
**Periapical Radiographs**											
	**Estimate**	***p*** **-Value**	**CILB**	**CIUB**							
ROC AUC Before DL–After DL	0.031	0.243	−0.022	0.085							
	R1	R2	R3	R4	R5	R6	R7	R8	R9	R10	Averaged ROC AUC
Before DL	0.775	0.870	0.850	0.838	0.811	0.898	0.829	0.877	0.717	0.845	0.831
After DL	0.911	0.925	0.863	0.891	0.856	0.809	0.838	0.867	0.766	0.898	0.862
**Bitewing Radiographs**											
	**Estimate**	***p*** **-Value**	**CILB**	**CIUB**							
ROC AUC Before DL–After DL	−0.009	0.746	−0.065	0.047							
	R1	R2	R3	R4	R5	R6	R7	R8	R9	R10	Averaged ROC AUC
Before DL	0.824	0.858	0.817	0.854	0.824	0.886	0.765	0.865	0.723	0.794	0.821
After DL	0.854	0.848	0.791	0.816	0.803	0.800	0.681	0.850	0.852	0.826	0.812

Abbreviations: AUC, area under the curve; CILB, 95% confidence interval lower bound; CIUB, 95% confidence interval upper bound; DL, deep learning; R, reader; ROC, receiver operating characteristic.

## Data Availability

The data that support the findings of this study are available from the corresponding author upon reasonable request.
